# Factors affecting age-appropriate timeliness of vaccination coverage among children in Lebanon

**DOI:** 10.12688/gatesopenres.12898.1

**Published:** 2018-12-16

**Authors:** Ziad Mansour, Racha Said, Lina Brandt, Joseph Khachan, Alissar Rady, Kamal Fahmy, M. Carolina Danovaro-Holliday

**Affiliations:** 1 Connecting Research to Development, Beirut, Lebanon; 2 World Health Organization Lebanon Country Office, Beirut, Lebanon; 3 World Health Organization Eastern Mediterranean Region Office, Cairo, Egypt; 4 World Health Organization, Geneva, Switzerland

**Keywords:** routine vaccination, survey, age-appropriate vaccination, timeliness, vaccination coverage

## Abstract

**Background:** The effect of immunization does not only depend on its completeness, but also on its timely administration. Routine childhood vaccinations schedules recommend that children receive the vaccine doses at specific ages. This article attempts to assess timeliness of routine vaccination coverage among a sub-sample of children from a survey conducted in 2016.

**Methods:** This analysis was based on data from a cross-sectional multistage cluster survey conducted between December 2015 and June 2016 among caregivers of children aged 12-59 months in all of Lebanon using a structured survey questionnaire. The analysis used Kaplan–Meier curves and logistic regression to identify the predictors of age-appropriate immunization.

**Results:** Among the 493 randomly selected children, timely administration of the third dose of polio vaccine, diphtheria-tetanus-pertussis (DTP)-containing vaccine and hepatitis B (HepB) vaccine occurred in about one-quarter of children. About two-thirds of children received the second dose of a measles-containing vaccine (MCV) within the age interval recommended by the Expanded Programme on Immunization (EPI). Several factors including socio-demographic, knowledge, beliefs and practices were found to be associated with age-appropriate vaccination; however, this association differed between the types and doses of vaccine. Important factors associated with timely vaccination included being Lebanese as opposed to Syrian and being born in a hospital for hepatitis B birth dose; believing that vaccination status was up-to-date was related to untimely vaccination.

**Conclusions:** The results suggest that there is reason for concern over the timeliness of vaccination in Lebanon. Special efforts need to be directed towards the inclusion of timeliness of vaccination as another indicator of the performance of the EPI in Lebanon.

## Introduction

Full immunization coverage is one of the key public health measures to prevent morbidity and mortality worldwide ^1^. Yet, the effect of immunization does not only depend on its completeness but also on its timely administration ^2^. If a child is not immunized appropriately or if immunization is delayed, then the child is at a higher risk of falling sick from a preventable disease ^3^. According to the World Health Organization (WHO), an invalid vaccine dose is defined as any dose of vaccine administered earlier than the recommended age ^4^. Risk factors for a child to receive untimely vaccine doses can be multifold and may relate to existing immunization practices, logistical aspects of providing vaccines, perceived contraindications, beliefs and attitudes towards vaccines or socioeconomic determinants ^5– 8^. Demographic characteristics, including the age, educational level and employment status of children’s caregiver, can be crucial for a child receiving timely vaccination or not ^9, 10^.

Demographics in Lebanon are dynamic and underlie constant changes due to the social, economic and political fluctuations inside and outside the country ^11^. The influx of Syrian refugees is the most recent shift, impacting on various spheres of life of the Lebanese population ^12^. With regard to immunization coverage, the demand for vaccines increased greatly as nationwide vaccination campaigns were introduced to ensure optimal coverage levels and to prevent disease outbreaks ^13^. Despite the prominent resilience of the Lebanese health care system, low immunization rates among Syrian refugee children persisted and are indicative of deficiencies in managing routine vaccination coverage during the crisis ^14, 15^. Furthermore, the variety of providers offering vaccines, as well as the multiplicity of schedules used, may result in different practices around the timeliness and simultaneity of vaccination.

Few studies exist that show how knowledge and awareness among parents influence a child’s immunization status in Lebanon ^16^ and no study on vaccination timeliness exists. Therefore, it is crucial to extend our understanding about attitudes and behaviors to establish evidence-informed interventions. In the region, studies have shown that parental knowledge and practices are associated with complete vaccination ^10^. In addition, the integration of immunization services in antenatal care may enhance vaccination take-up and timely administration ^17^.

In 2016, a district-based immunization coverage cluster survey was conducted in Lebanon following the Syrian crisis; it included potential determinants for vaccination ^18^. In order to further understand gaps in vaccine administration, this study analyzed vaccination timeliness and adherence to the national vaccination schedule set by the Ministry of Public Health (MoPH). Furthermore, key socio-demographic, knowledge, belief and practice factors associated with timely vaccination among Lebanese and Syrian children aged 12–59 months old were investigated.

## Methods

### Survey background

A national cross-sectional survey was conducted among caregivers of children aged 12–59 months following a stratified cluster sampling design. The survey was implemented from December 2015 to June 2016 in all districts of Lebanon with the exception of Nabatieh due to inaccessibility and was designed to provide district-based vaccine coverage estimates ^18^.

### Sampling

The original study population included 10,140 children from 26 districts in Lebanon, irrespective of their nationality. Population estimates obtained from the Central Administration of Statistics and United Nations High Commissioner for Refugees ^19, 20^ were used to randomly select 26 clusters in each district with probability proportionate to estimated size, following the 2005 WHO cluster evaluation survey methodology ^4^; there were no up-to-date sampling frames that would have allowed conducting true probability sampling. In total, 15 children were selected by team supervisors from each cluster using a systematic random approach, with each child being selected from a different household during in-person house visits ^4^. The teams were trained to adhere to the protocol and avoid changing pre-selected households. For this study, a sample of 500 children (a size arbitrarily defined by the study team) was randomly selected among the 3,728 children with documented vaccination in the main survey, i.e., who had a) pictures of the vaccination card available and b) at least one available date of vaccine administration on the card as age at vaccination was needed for this study. Children were excluded from this random selection if they had vaccination cards without any registered vaccination date or without a reported date of birth or if they were neither Lebanese nor Syrian.

### Outcome variables

Any child without evidence of having received specific vaccine doses from the vaccination card was considered as not vaccinated. Children for whom no evidence was found on the date of vaccination for an antigen were excluded from the analysis related to this specific antigen. To assess delays in vaccination, the recommended vaccination schedule for children less than 5 years old, adopted by the Lebanese MoPH, was used ^21^.

Timeliness was assessed through the age-appropriate vaccination coverage, defined as per the criteria in Table 1. These timely criteria included having received hepatitis B (HepB) birth dose in the first 72 hours after birth, three pentavalent and polio vaccines, the first dose starting from 60 days and the last one up to 195 days ^1^, two measles-containing vaccines (MCV), the first dose starting from 270 days and the last one up to 555 days, and with an interval of at least 28 days between subsequent doses containing the same antigen.

**Table 1.** Recommended ages for routine vaccination for children aged 0–59 months in Lebanon.

Vaccine	Birth dose	First dose	Last dose
Hepatitis B	First 72 hours (3 days)	30 to 75 days	180–195 days
Polio	NA	60–75 days	180–195 days
Diphtheria, Tetanus, Pertussis	NA	60–75 days	180–195 days
Measles	NA	270–300 days	NA
Measles, Mumps, Rubella	NA	If child received measles → 360–465 days (MCV schedule 1)	NA
		If child didn’t receive measles → 360–390 days (MCV schedule 2)	540–555 days

* In this article, measles and MMR vaccines were entered as measles-containing vaccine (MCV) (either measles or MMR vaccines).

### Data analysis

Data was analyzed using Stata software, version 14, applying the “svyset” command for complex survey designs. National estimates took into account the sampling design (stratum, district and governorate-specific weight) in order to ensure that each selected child represents a certain number of similar eligible children from the population.

Timeliness for all antigens was calculated for the sampled Lebanese and Syrian children separately with 95% confidence intervals (CI). The period of time until a child received a vaccine dose was calculated by subtracting the birth date from the date of vaccination. For each vaccine, the cumulative probability of being vaccinated at age t was estimated by inverse Kaplan–Meier survival function, or 1−SKM(t) ^22^. For analysis purposes, months were considered as having 30 days.

In addition to vaccination data, information on children’s socio-demographic characteristics and respondents’ knowledge, beliefs and practices related to vaccination were collected using a structured questionnaire ^23^ administered by trained interviewers. An analysis of risk factors was conducted for non-age-appropriate vaccination. Using age-appropriate vaccination as the main outcome, crude and adjusted odds ratios (OR) were calculated by applying univariable and multivariable logistic regression models, respectively. All the independent variables significantly associated with the outcome at univariable analysis with a cut-off level at p-value < 0.2 were included initially in a multivariable model. The significance was set at 5% (p-value <0.05).

### Ethical considerations

Before starting the interview with caregivers of eligible children, oral informed consent was obtained. Written consent was not obtained as this is not a common practice for this type of studies in Lebanon, given the low levels of literacy among certain populations and the non-sensitive nature of the information obtained. Confidentiality was strictly applied during all study procedures, including the storage of vaccination cards and the final database. Ethical approval was attained from the Institutional Review Board at Sagesse University as per the reference number IRB120416B.

## Results

Of the 500 randomly selected children, seven who were neither Lebanese nor Syrians were excluded, leading to a final sample for analysis of 493 children. Their characteristics are described in Table 2.

**Table 2.** Sample characteristics (unweighted).

Characteristics	Subjects	
	Number	Percentage
Nationality of the child		
Lebanese	393	79.7
Syrian	100	20.3
Gender of the child		
Male	267	54.2
Female	226	45.8
Age of the child, months		
12–23	156	31.6
24–35	139	28.2
36–47	111	22.5
48–59	87	17.7
Mother’s educational status		
No formal education	63	12.8
Primary/Complementary level	135	27.4
Secondary/Post school technical level	170	34.5
University level	120	24.3
Doesn’t know/Refused to answer	5	1.0

### Age-appropriate vaccination coverage (timeliness)

**Figure 1.**
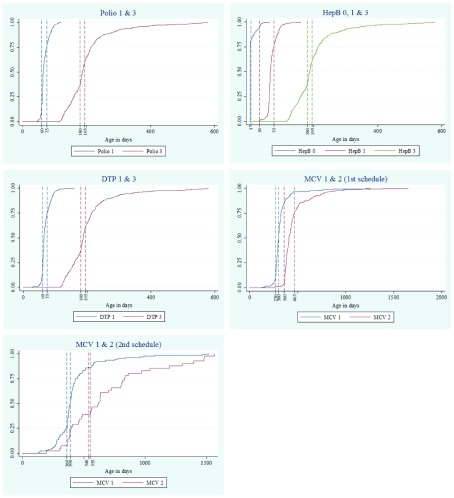
Inverse Kaplan–Meier curves showing the proportion of children vaccinated with each vaccine’s dose. For each dose, two reference lines are drawn to mark the age-appropriate intervals for vaccination.

The inverse Kaplan–Meier survival curves depict the vaccination coverage of polio, HepB, diphtheria-tetanus-pertussis (DTP) and MCV at different ages (in days) after birth, as presented in Figure 1. HepB vaccination coverage within 72 hours following birth was 78.3% (95% CI: 72.7-83.1). The vaccination coverage for the third dose of polio was 64.3% (95% CI: 58.4-69.8) by 6.5 months (195 days) of age. A similar picture of vaccination coverage was presented for the third doses of HepB 66.1% (95% CI: 60.3-71.4) and DTP 65.3% (95% CI: 59.6-70.7) following 195 days. The coverage for the second dose of MCV at 15.5 (465 days) and 18.5 (555 days) months of age was calculated to be 75.2% (95% CI: 67.8-81.3) and 37.9% (95% CI: 22.4-53.4), respectively. Later doses in a series (i.e., 3 ^rd^ DTP, polio and HepB) were more likely to be delayed than first doses. This is particularly striking for the second dose of measles, mumps and rubella (MMR) when children received MMR as their first MCV dose (schedule 2).

Age-appropriate vaccination coverage estimates for the first and third doses of polio, HepB, DTP and MCV vaccines, in addition to HepB birth dose, are displayed in Table 3, by nationality and age cohort. Timely vaccination ranged from 27.1% (95% CI: 21.8-33.1) for the third dose of polio to 82.8% (95% CI: 77.4-87.1) for HepB at birth among Lebanese children. Timely receipt of vaccinations fluctuated between 19.3% (95% CI: 10.7-32.5) for the third dose of DTP and 61.2% (95% CI: 46.8-73.8) for the first dose of HepB among Syrian children. A statistically significant difference was observed between Lebanese and Syrian children for the first doses of polio, HepB, DTP and MCV, in addition to HepB birth dose. Lebanese children showed higher proportions of age-appropriate vaccination than Syrian children. Moreover, vaccination coverage within the appropriate age among Lebanese was the highest for children aged 24–35 months for all vaccine doses, excluding HepB birth dose. No clear pattern on timeliness by age cohort was seen.

**Table 3.** Timely vaccination for Lebanese and Syrian children in each age group.

Type of vaccine	Total		Age groups							
	12–59 months		12–23 months		24–35 months		36–47 months		48–59 months	
	Lebanese	Syrian	Lebanese	Syrian	Lebanese	Syrian	Lebanese	Syrian	Lebanese	Syrian
	Percentage (95%CI)	Percentage (95%CI)	Percentage (95%CI)	Percentage (95%CI)	Percentage (95%CI)	Percentage (95%CI)	Percentage (95%CI)	Percentage (95%CI)	Percentage (95%CI)	Percentage (95%CI)
Polio 1 ^st^ dose	66.4 (59.7-72.4)	53.1 (38.8-67.0)	63.3 (51.3-73.8)	54.0 (32.9-73.8)	69.7 (57.9-79.4)	61.5 (36.2-81.9)	66.0 (52.2-77.5)	49.4 (23.7-75.5)	67.6 (53.7-79.0)	38.0 (13.5-70.6)
Polio 3 ^rd^ dose	27.1 (21.8-33.1)	19.7 (10.8-33.3)	29.0 (20.1-39.9)	21.0 (7.1-48.0)	33.6 (24.3-44.3)	20.6 (7.9-43.9)	20.6 (12.1-32.8)	1.2 (0.2-9.1)	17.7 (8.5-33.3)	42.1 (13.8-76.9)
DTP 1 ^st^ dose	66.0 (59.6-71.8)	53.0 (38.9-66.6)	62.8 (50.8-73.4)	54.0 (32.9-73.8)	68.7 (57.3-78.2)	59.9 (35.4-80.3)	66.5 (52.8-77.9)	55.2 (29.3-78.5)	67.6 (53.7-79.0)	29.1 (8.7-63.8)
DTP 3 ^rd^ dose	26.7 (21.5-32.6)	19.3 (10.7-32.5)	27.9 (19.1-38.7)	21.7 (7.5-48.6)	34.5 (25.4-45.0)	20.5 (7.9-43.6)	19.5 (11.4-31.2)	1.8 (0.4-8.0)	17.7 (8.6-33.0)	46.6 (15.5-80.6)
HepB birth dose	82.8 (77.4-87.1)	57.8 (40.0-73.7)	74.3 (63.1-83.0)	61.3 (26.5-87.5)	83.6 (72.7-90.8)	37.9 (18.0-63.0)	86.1 (74.0-93.1)	76.6 (47.3-92.3)	96.1 (89.9-98.6)	58.9 (25.9-85.4)
HepB 1 ^st^ dose	80.2 (75.0-84.6)	61.2 (46.8-73.8)	73.9 (63.2-82.4)	56.2 (34.7-75.6)	89.8 (81.9-94.4)	64.6 (38.5-84.2)	81.4 (70.0-89.1)	74.0 (47.6-89.9)	76.3 (62.0-86.3)	38.0 (13.5-70.6)
HepB 3 ^rd^ dose	27.6 (22.3-33.6)	19.9 (11.0-33.3)	28.9 (20.0-39.8)	21.7 (7.5-48.6)	34.8 (25.5-45.4)	20.5 (7.9-43.6)	19.8 (11.5-32.0)	1.8 (0.4-8.0)	20.0 (10.3-35.4)	60.3 (20.8-89.7)
MCV 1 ^st^ dose	44.3 (38.1-50.7)	30.7 (19.7-44.4)	50.5 (37.8-63.1)	45.5 (24.3-68.4)	47.0 (34.7-59.5)	15.3 (4.3-41.9)	38.4 (25.0-53.7)	27.6 (7.6-64.0)	34.8 (22.4-49.6)	35.1 (9.1-74.6)
MCV 2 ^nd^ dose	63.5 (55.4-70.9)	57.8 (42.4-71.8)	84.3 (70.6-92.4)	86.3 (64.0-95.7)	60.9 (45.7-74.2)	34.0 (14.1-61.9)	57.1 (40.2-72.5)	47.2 (14.4-82.7)	48.0 (33.5-62.8)	63.3 (19.7-92.4)

CI, confidence interval; DTP, diphtheria-tetanus-pertussis; HepB, hepatitis B; MCV, measles-containing vaccine.

### Factors associated with being vaccinated at the appropriate age

The results of the univariate and multivariable models for factors associated with age-appropriate vaccination are presented in Table 4. Syrian children were less likely to be vaccinated overall, and on time. Adjusting for all variables in the model, the odds of timely receipt of the first doses of polio (OR: 2.5; 95% CI: 1.1-5.4), DTP (OR: 2.4; 95% CI: 1.1-5.4) and HepB (OR: 4.0; 95% CI: 1.1-14.1) vaccines were higher among children whose parents expressed the unlikelihood of their children becoming sick if they were not immunized. Age-appropriate vaccination with HepB birth dose was more likely if the child was vaccinated in a place other than a health facility or private clinic, mainly in hospitals (OR: 2.9; 95% CI: 1.1-8.1). The odds of receipt of the third dose of DTP vaccine by 195 days were 1.9 times higher among children whose caregivers knew the number of times the child should be taken for vaccination to complete all vaccines before one year of age (OR: 1.9; 95% CI: 1.1-3.7). This association was also significant for the third dose of HepB vaccine (OR: 2.0; 95% CI: 1.1-3.8). Believing that vaccination status was up-to-date for the child’s age was related to untimely vaccination with the first dose of MCV (OR: 0.1; 95% CI: 0.0-0.7).

**Table 4.** Odds ratio of socio-demographic, vaccination knowledge, beliefs and practices factors significantly associated with timely vaccination.

Variables ^a^	Type of vaccine								
	Polio		DTP		Hepatitis B			MCV	
Dose	1 ^st^	3 ^rd^	1 ^st^	3 ^rd^	0	1 ^st^	3 ^rd^	1 ^st^	2 ^nd^
Odds Ratio	AOR ^b^ (95%CI) Crude OR (95%CI)	AOR ^b^ (95%CI) Crude OR (95%CI)	AOR ^b^ (95%CI) Crude OR (95%CI)	AOR ^b^ (95%CI) Crude OR (95%CI)	AOR ^b^ (95%CI) Crude OR (95%CI)	AOR ^b^ (95%CI) Crude OR (95%CI)	AOR ^b^ (95%CI) Crude OR (95%CI)	AOR ^b^ (95%CI) Crude OR (95%CI)	AOR ^b^ (95%CI) Crude OR (95%CI)
Nationality of the child									
Lebanese (Ref)									
Syrian					**0.2 (0.1-0.4)** 0.3 (0.1-0.7)	**0.3 (0.2-0.7)** 0.4 (0.2-0.8)			
Age of the child, months									
12–23 (Ref)									
24–35						**2.1 (1.1-4.5)** 2.3 (1.1-4.6)			**0.2 (0.1-0.5)** 0.2 (0.1-0.5)
36–47									**0.2 (0.1-0.5)** 0.2 (0.1-0.5)
48–59					**3.7 (1.4-9.5)** 3.4 (1.4-8.4)				**0.2 (0.1-0.4)** 0.2 (0.1-0.4)
Respondent believes that child’s vaccination is up-to-date for his/her age									
No (Ref)									
Yes								**0.1 (0.0-0.7)** 0.2 (0.0-0.7)	
Place where child was vaccinated during the last visit for vaccination									
Health facility/PHC (Ref)									
Private clinic									
Other (hospitals, etc.)					**2.9 (1.1-8.1)** 3.1 (1.2-7.9)				
Respondent knows the number of times the child should be taken for vaccination to complete all vaccines before reaching 1 year of age									
No (Ref)									
Yes				**1.9 (1.1-3.7)** 2.5 (1.4-4.6)			**2.0 (1.1-3.8)** 2.5 (1.4-4.5)		
Likelihood of child becoming sick if he/she is not immunized according to respondent’s belief									
Extremely likely/likely (Ref)									
Neutral/Extremely unlikely/Likely	**2.5 (1.1-5.4)** 2.0 (0.8-4.8)		**2.4 (1.1-5.4)** 2.0 (0.8-4.9)			**4.0 (1.1-14.1)** 2.6 (0.8-9.2)			
Main source of information to decide about vaccinating your child used by respondent									
Private physician/Health facility staff (Ref)									
Media									
Nursery/School					**0.1 (0.0-0.8)** 0.2 (0.1-0.7)		**0.1 (0.0-0.9)** 0.2 (0.1-1.0)		

^a^Only variables significantly associated with the outcome are reported. ^b^Adjusted for all other variables included in the model. AOR, adjusted odds ratio.

There were no significant differences in age-appropriate vaccination based on children’s gender and mothers’ education. Similarly, taking the vaccination card when visiting the doctor or health facility for immunization, registering the administered vaccine on the vaccination card, receiving advice on next vaccination date, making decisions about vaccinating the child, knowing that vaccines are given free of cost in the public sector and knowing that the vaccination status is checked when starting school or kindergarten were not found to be significantly associated with timely vaccination.

## Discussion

This study is the first to assess the age-appropriate vaccination coverage and its predictors among a subset of Lebanese and Syrian children aged 12–59 months residing in the Lebanese communities in 2016. Vaccination programs aim at attaining the highest level of protection against vaccine-preventable diseases at a young age together with high immunization coverage rates ^24^. Routine vaccination timeliness and completeness are still public health challenges. Evaluating age-appropriate vaccination provides valuable insights even in populations with very high up-to-date immunization coverage ^9^. Currently, timeliness is not routinely used as an indicator to evaluate immunization programs in Lebanon. Special efforts need to be directed towards the inclusion of timeliness of vaccination as another indicator of the performance of the Expanded Programme on Immunization in Lebanon considering its crucial role in improving children’s health and survival and reducing the risk of disease in children ^25^.

In this study, the age-appropriate vaccination coverage has been described and graphically visualized using the inverse Kaplan–Meier survival curve. About 75% of children, among those included in this survey sub-sample, received the first doses of polio and DTP vaccines by 75 days, but few were immunized with these vaccines at the recommended age. The situation was even more worrisome in the case of the third doses of polio, HepB and DTP vaccines; while about 65% of children received these doses by 195 days; only 1 in 4 received them at the appropriate age. The doses of MCV vaccine were more timely than the third doses of polio, HepB and DTP, which can be because MCV is a two-dose series and has a longer interval between the doses. Despite the efforts made to ensure MCV timeliness, measles and mumps outbreaks emerged in Lebanon in early 2018, which may be attributed to the challenge of having a large influx of Syrian refugees ^11, 26^. With the existence of such a pool of vulnerable children, outbreaks may occur much faster when untimely vaccination is coupled with low immunization coverage and reduced vaccine effectiveness ^27^. The difference in vaccination schedules and the application of different vaccines or vaccine combinations may also contribute to the variation in timeliness ^28^. The results of this analysis are in line with the results of previous studies in other countries that showed relatively low proportions of children vaccinated at the appropriate age ^29, 30^.

This study identified several factors, including socio-demographic, knowledge, beliefs and practices associated with age-appropriate vaccination; however, this association differed between the types and doses of vaccine. First, receiving HepB vaccine birth dose from the hospitals was a positive predictor for age-specific vaccination, compared to those who received vaccination in a health facility. This may reflect that health services, mainly having a better utilization of vaccination services, were more accessible by mothers who gave birth at a hospital, especially for HepB birth dose to be given within 3 days of birth ^31^. Second, erroneously believing that children’s vaccination is up-to-date for their age was negatively associated with the administration of timely vaccines. A mother’s communication with health providers might be hindered by a low education level influencing negatively the immunization through the wrong beliefs ^28, 32, 33^. Poor knowledge of immunization schedules among mothers may also explain this negative association. Third, using the information provided by schools and nurseries to decide about the child’s vaccination rather than obtaining this information from a private physician or health facility staff was also a risk factor. Healthcare facilities and private clinics are responsible to a greater extent for the correct administration of vaccinations. These providers are expected to play an important role in determining invalid vaccinations ^34^. Further studies are needed to examine the factors associated with vaccination timeliness in Lebanon, as factors related to timely vaccination were not identified for all vaccine doses.

Our study was subjected to important limitations. First, the timeliness assessment was done on a small sub-sample of 493 children, because children’s vaccine status was assessed case by case. Only children with available vaccination cards with administration dates were included as this was the only way to obtain accurate information on age at vaccination. While the exclusion of caregiver's recall reduced recall bias, purely relying on vaccination cards may have resulted in an underestimation of vaccination delay, particularly those children without an available vaccination card might be more prone to untimely vaccination. Another obstacle during the assessment was the illegible cards. Although vaccination cards were assumed to have the most valid reporting of received vaccination, the handwriting of health workers created difficulty to accurately determine the date of vaccine administration. Second, the definition of timeliness was specifically developed for this assessment based on expert opinion because diverse practices exist among providers in the Lebanese vaccine system. The diversity in practice can be related to different available vaccination schedules and vaccines or vaccine combinations. These factors jeopardize the generalizability and comparability of results to other assessments, and interpretations should always consider the context of this study. Despite these limitations, our study is the first one to show that untimely vaccination is of concern in Lebanon, and to shed light on factors related to timely vaccination that could be useful when devising strategies to improve age-appropriate vaccination.

## Data availability

### Underlying data

Owing to data protection concerns, the datasets generated and analyzed during the study are not publicly available. Readers can access data by contacting the corresponding author at saidr@crdconsultancy.org. Data will only be shared with researchers for the purpose of reanalysis and grant proposals.

### Extended data

The questionnaire used in the study is available on figshare. DOI: https://doi.org/10.6084/m9.figshare.7454870 ^23^.

This is available under the terms of the Creative Commons Zero "No rights reserved" data waiver (CC0 1.0 Public domain dedication).

## Footnotes

The first dose of Hepatitis B vaccine was considered timely if given starting from 30 days.
