# Supplementary material for: Factors affecting age-appropriate timeliness of vaccination coverage among children in Lebanon
Source: Gates Open Res. 2018 Dec 16;2:71. [Version 1] doi: 10.12688/gatesopenres.12898.1 (PMC6362301; doi:10.12688/gatesopenres.12898.1)
Supplement: Peer review history

# Peer review: Reviewer response for version 1

**DOI:** 10.21956/gatesopenres.13994.r26872

**Published:** 2019-01-30

**Authors:** Mark A. McKinlay

**Affiliations:** 1 Center for Vaccine Equity (CVE) , The Task Force for Global Health, Decatur, GA, USA

This well designed and analyzed study analyzed for the first time identifies the factors affecting age-appropriate vaccination of children in Lebanon. The authors clearly and concisely present the background for the study and identified the striking findings of the low percentage of children in Lebanon that are being vaccinated (polio third dose only 64.3%) in some cases and the even lower percentage receive the vaccine in a timely manner (27.1% for 3 ^rd^ dose of polio). These results will hopefully provide the evidence needed to improve the timeliness of vaccination in the country. The identified key factors impacting the rates of age-appropriate vaccination should be helpful in implementing corrective activities to improve performance. The parent’s misunderstanding around the vaccination schedule is concerning and not seeking the information on schedules from a physician or health facility staff needs to be addressed.

 It is recommended that this article be indexed.

I have read this submission. I believe that I have an appropriate level of expertise to confirm that it is of an acceptable scientific standard.

# Peer review: Reviewer response for version 1

**DOI:** 10.21956/gatesopenres.13994.r26835

**Published:** 2019-01-09

**Authors:** Asnakew Tsega

**Affiliations:** 1 JSI and Maternal and Child Survival Program (MCSP), Washington, DC, USA

**General comment**

 The paper is on factors affecting age-appropriate timeliness of vaccination coverage among children and it is a cluster coverage survey. The survey was well designed and appropriately implemented and the results presented clearly. Many surveys don't address the timeliness of vaccination and this fills this gap.

**Specific comments**

 The timeliness of vaccination was defined as DPT containing vaccine 1st dose 60-75 days and 3rd dose 180-195 days as valid doses. However, the WHO reference manual mentioned by authors as reference 4 states the following: Valid dose "immunizations given when the child was the appropriate minimum age i.e 60 days based on Lebanon schedule and if the immunization is one of a series that they were given after an appropriate minimum interval of time of 28 days." Therefore, the two conditions for a valid dose are a minimum age and a minimum interval. There is no maximum age or maximum interval. The authors should consider reviewing the age of vaccination based on the WHO guidance i.e no need to limit the maximum age for 1st dose at 75 days and the 3rd dose at 195 days (6.5 months).

I have read this submission. I believe that I have an appropriate level of expertise to confirm that it is of an acceptable scientific standard.
